# Bidirectional associations between rheumatoid arthritis and depression: a nationwide longitudinal study

**DOI:** 10.1038/srep20647

**Published:** 2016-02-09

**Authors:** Ming-Chi Lu, How-Ran Guo, Miao-Chiu Lin, Hanoch Livneh, Ning-Sheng Lai, Tzung-Yi Tsai

**Affiliations:** 1 Division of Allergy, Immunology and Rheumatology, Dalin Tzuchi Hospital, The Buddhist Tzuchi Medical Foundation, 2 Minsheng Road, Dalin Township, Chiayi 62247, Taiwan; 2 School of Medicine, Tzu Chi University, 701 Jhongyang Road Section 3, Hualien 97004, Taiwan; 3 Department of Environmental and Occupational Health, National Cheng Kung University Hospital, College of Medicine, National Cheng Kung University, 138 Sheng-Li Road, Tainan 70428, Taiwan; 4 Department of Occupational and Environmental Medicine, National Cheng Kung University Hospital, 138 Sheng-Li Road, Tainan 70428, Taiwan; 5 Occupational Safety, Health, and Medicine Research Center, National Cheng Kung University, 138 Sheng-Li Road, Tainan 70428, Taiwan; 6 Department of Nursing, Dalin Tzuchi Hospital, The Buddhist Tzuchi Medical Foundation, 2 Minsheng Road, Dalin Township, Chiayi 62247, Taiwan; 7 Rehabilitation Counseling Program, Portland State University, Portland OR 97207-0751, USA; 8 Department of Medical Research, Dalin Tzuchi Hospital, The Buddhist Tzuchi Medical Foundation, 2 Minsheng Road, Dalin Township, Chiayi 62247, Taiwan; 9 Department of Nursing, Tzu Chi University of Science and Technology, 880 Chien-Kuo Road Section 2, Hualien 62247, Taiwan

## Abstract

Rheumatoid arthritis (RA) and depression may be associated with each other pathophysiologically, but few studies have been conducted on the interplay between these two diseases using longitudinal measurement. Therefore, we used the National Health Insurance Research Database of Taiwan to investigate the bidirectional associations between RA and depression. One cohort was included to analyze RA predicting the onset of depression and a second cohort for analysis of depression predicting RA. A sex- and age-matched control group was included for both. The incidence of depression in RA subjects was higher than in non-RA subjects [15.69 vs. 8.95 per 1,000 person-years (PYs)], with an adjusted hazard ratios (HRs) of 1.69 [95% confidence interval (CI), 1.51–1.87]. The incidence of RA was higher in depressed than non-depressed individuals (2.07 vs. 1.21 per 1,000 PYs), with an adjusted HRs of 1.65 (95% CI, 1.41–1.77). This population-based cohort study suggested strong bidirectional relationships between RA and depression. Healthcare providers are recommended to facilitate the implementation of more effective therapeutic interventions to achieve favorable prognosis, especially for those with new-onset or younger cases.

Although the possible mechanisms linking psychiatric disorders and rheumatoid arthritis (RA) still remain unclear^1^, some studies reported that a clear clustering of depressive symptoms among patients with RA^2^,^3^,^4^,^5^. Notably, those RA patients suffering from concomitant depression had nearly a 7.2% increase in medical costs per year ($12,225 vs. $11,404)^6^, and more than doubled the likelihood of mortality compared to patients with RA only^7^, suggesting that prompt provision of psychosocial support is of utmost importance.

The comorbid relationship may imply a putative causal link between RA and depression, possibly through a dysfunctional neuroendocrine system^2^,^8^,^9^. Some empirical reviews further demonstrated that intracellular signaling pathways, such as PI-3K/AKT/mTOR and stress- and mitogen-activated protein kinases (SAPK/MAPK), may provide a connection between the two diseases^10^,^11^,^12^. However, unlike studies on the risk of depression in patients with RA^3^,^4^,^5^,^13^, empirical research examining whether there is also an elevated risk of RA in patients with depression are sparse and the results are conflicting. A community survey involving 7,076 subjects found no relationship between the occurrence of depression and the predisposition of rheumatologic disorders^14^, whereas two other studies found that depression was associated with a greater-adjusted risk of arthritis onset as compared to those in which this condition was absent^15^,^16^.

Given the corresponding health care burden and adverse clinical manifestations of these medical conditions, the understanding of the bidirectional relationships between the RA and depression has become a pressing issue, and would be of great importance in setting prevention efforts and priorities for reducing the presence of concomitant depressive symptoms among RA subjects, and RA risk among patients with depression. To our knowledge, to date, only one study has conducted such analyses which were based on these populations^14^. This study, however, was limited due to the cross-sectional design and self-administered questionnaire, which could be different from clinical diagnosis and confounded by recall bias. To address this concern, we applied claim data from the National Health Insurance (NHI) of Taiwan to better understand the risk of developing RA among patients with depression and the risk of developing depression among RA patients.

## Results

### First Analysis: RA and the Subsequent Risk of Depression

Table 1 shows the basic characteristics of the RA and non-RA cohorts. We established a cohort of 8,331 RA patients and a non-RA cohort of 15,456 subjects. Age and sex distributions were similar for both cohorts with a mean age of approximately 54 years of age, and a majority (over 2/3) of female patients in both groups. RA cohorts were more likely to reside in a rural area (*P* < 0.01) and suffer from some common comorbidities, including hypertension, heart disease, stroke, chronic kidney disease, diabetes, and alcohol dependence syndrome (all *P* < 0.01).

In all subjects, a total of 2,033 episodes of depression occurred, which consisted of 958 among RA cohorts and 1,075 among the non-RA cohorts during 61,075.48 and 12,100.95 PYs of follow-up, respectively. Incidence of depression in RA cohorts was higher than in the non-RA cohorts (15.69 vs. 8.95 per 1,000 PYs), with an adjusted HRs of 1.69 [95% confidence interval (CI), 1.51–1.87] as shown in Table 2. Additionally, among male and female cohorts with RA, the adjusted HRs of depression during the 14-year follow-up period were, respectively, 1.65 and 1.73 for the risk of depression. The hazards were comparable between men and women (*P* = 0.78). On the other hand, regardless of gender, the age-stratified analysis revealed that age significantly interacted with RA on the risk of depression, in which the higher adjusted HRs were observed in both female and male RA cohorts aged≤40 years (2.14; 95% CI, 1.62–2.69 and 2.76; 95% CI, 1.83–4.59, respectively). The interactions were confirmed by further analyses showing that the interaction term of age and RA was statistically significant in both sexes (all *P *< 0.05). Analysis stratified by the time from RA diagnosis showed that the higher incidence rates of depression in the RA cohorts, as compared to the non-RA cohorts, occurred within two years since RA diagnosis, with adjusted HRs of 1.98 (95% CI, 1.43–2.15).

### Second Analysis: Depression and the Subsequent Risk of RA

The distributions of baseline characteristics in the depression and non-depression cohorts are summarized in Table 3. Mean age of cohorts with depression was 48.26, similar to that of the non-depression cohorts of 48.16. We found that the depression cohorts were more likely to reside in less urbanized area (*P* < 0.01) and had comorbidities such as hypertension, heart disease, stroke, chronic kidney disease, diabetes, cancer, alcohol dependence syndrome, and tobacco use (all *P* < 0.01).

Table 4 reports the occurrences of RA over the 14-year follow-up period for the two cohorts. At the end of follow-up, there were 1,555 incident RA cases, including 718 from the depression cohorts and 837 from the non-depression cohorts. The incidence of RA was significantly higher in the depression cohorts than in non-depression cohorts (2.07 vs. 1.21 per 1,000 PYs). Multivariate Cox proportional hazard regression model revealed that the subjects with depression had 65% higher risk of developing RA compared with those without depression (95% CI, 1.41–1.77). In both male and female groups, the finding indicated that depression was significantly related to the subsequent risk of RA. The incidence of RA was comparable between men and women, with adjusted HRs of 1.61 (95%CI, 1.28–2.02) and 1.55 (95% CI, 1.34–1.74), respectively (*P* = 0.91). The effects of age was statistically significant in both sexes, and the increased RA risk subsequent to depression was observed for subjects aged 40 or less, with adjusted HRs of 1.95 (95% CI, 1.37–2.48) in females and 2.04 (95% CI, 1.09–3.86) in males. The interactions between age and depression on RA were confirmed by further analyses showing that the interaction term was statistically significant in both sexes (*P* < 0.01 in both). As to the risk in relation to time since depression onset, the higher incidence rate of RA in the depression cohorts, as compared to non-depression cohorts, was observed in the 3–5 and >5 year periods after onset of depression, with adjusted HRs of 1.83 (95% CI, 1.61–2.19) and 1.58 (95% CI, 1.34–1.79), respectively.

## Discussion

To the best of our knowledge, this is the first evidence-based cohort study applying a large nationwide claims-based data to address the bidirectional relationships between RA and depression, allowing for more potent validation of findings concerning the temporal association between these two diseases.

This 14-year follow-up study suggests that RA cohorts have nearly 70% higher risk of depression than non-RA cohorts. This finding is consistent with earlier research and adds to the growing body of literatures on this topic^3^,^4^,^5^,^13^,^14^. Furthermore, based on the stratified analysis for risk of depression subsequent to RA, we discovered that younger RA subjects, regardless of gender (aged 40 years or younger), carried the highest adjusted HRs for the risk of depression compared to non-RA controls of similar age, thus concurring with prior results^14^. A possible explanation for this finding is that most young RA subjects may be actively employed, and RA typically progresses and ultimately results in chronic functional disability. Therefore, this joint impact of physical limitations and work restrictions may negatively affect the individual and trigger negative affectivity, as indicated by higher level of depression^17^. In addition, we observed that a higher risk of depression occurred within 2 years after RA diagnosis. It was inferred that the newly-diagnosed patients might be psychologically fragile and overwhelmed by RA symptoms^18^. Therefore, future studies could assess the benefit of implementing a standardized care pathway, which closely evaluates psychological status among RA patients within the first years of diagnosis^2^,^3^,^19^.

Regarding the risk of RA onset subsequent to depression, our findings showed that subjects with depression are at an elevated risk of developing RA, echoing previous reports^15^,^16^. Furthermore, a sensitivity analysis, limited to the cases that were admitted to the hospitals due to the primary diagnosis of depression, also indicated that RA risk was slightly greater for inpatients with depression, with adjusted HRs of 1.75 (95% CI, 1.58–1.93). All of these results further supported the premise that the elevated level of proinflammatory cytokines emerging from psychological disorders may provoke chronic medical illnesses, such as RA, through protracted immune dysregulation^20^,^21^. The findings, however, are inconsistent with a former report^14^. This disparity can be explained by the difference in screening measures and design used in both studies. van’t Land and colleagues used a self-reported questionnaire to arrive at a psychiatric diagnosis of depression^14^, a procedure which may have obscured the findings due to recall and social desirability biases. Also, the cross-sectional design employed in that study does not allow for any inference about any cause-and-effect relationships between the tested variables.

With the exception of elderly subjects, our findings indicated that the total, and age- and sex- specific incidence rates of RA were consistently and significantly higher in the depression cohorts than in non-depression cohorts. We also observed that the risk of RA among depression patients was higher among subjects aged 40 years or younger. Two potential reasons may account for this finding. First, once subjects with a proinflammatory disposition have been diagnosed with depression, at younger age, they would be, subsequently, be at a higher risk of having RA, as compared to individuals who are older. Second, adults aged 40 years or younger are typically actively engaged in the labor market^22^, and accordingly, the combined stress of the workload and the need to cope with the clinical manifestations of depression, may insidiously aggravate the level of plasma concentrations of inflammatory cytokines, such as tumor necrosis factor-α (TNF-α), interleukin-1 (IL-1) or IL-6, thus increasing the vulnerability to RA^19^,^23^.

Although the current study provided support for an empirical link between RA and depression, the exact pathophysiological mechanisms involved in these two illnesses still remains elusive^1^. Neurotransmitter deregulation and dysfunctional intracellular signaling, however, was suggested as a link between psychological stress and immune-related disease. For example, exposure to a chronic negative mood has been related to dysfunction of the hypothalamic-pituitary-adrenal (HPA) axis and the peripheral release of glucocorticoids, along with increased activation of the sympathetic, as well as decreased activation of the parasympathetic branches of the autonomic nervous system, thereby inducing the expression of inflammatory markers, which in turn produce initiation and progression of autoimmune diseases^8^,^9^. Conversely, a growing body of evidence has also indicated that these proinflammatory cytokines, such as TNF-α and some interleukins, may mediate the HPA activation in response to various threats to homeostatic balance, and could, therefore, play a decisive role in the generation of major depression^24^,^25^.

Additionally, evidence has also supported the link between RA and depression through the activation of intracellular signaling pathway, such as SAPK/MAPK and PI-3K/AKT/mTOR. The SAPK/MAPK pathway was found to be related to the synovial hyperplasia and MMP gene expression, which may further degrade the collagen matrix components of the joints, thereby inducing a greater susceptibility of RA^10^,^26^. Both animal experiments and human studies have reported that MAPK had been increasingly recognized as an important regulator of the central nervous system (CNS), and was implicated in neuronal axonal development, inflammation and depression^27^,^28^. As to the influence of the PI-3K/AKT/mTOR pathway, it has been established that it not only causes the aggressiveness of immune cell and synoviocyte proliferation to provoke development of RA^10^, but also plays a crucial role in the function of dopamine, a neurotransmitter whose malfunctioning has been associated with a greater risk for reduced neuroprotection and the development of psychosis^12^.

Several important limitations of this study should be noted when interpreting the results. First, we could not account for several potential confounding factors, such as social networks, religious beliefs, or educational level because such data were unavailable in the LHID. Future research using those untested variables is needed to better assess if the present findings could be replicated across diverse groups of individuals. Second, the identification of exposure and outcome were based on ICD-9-CM, and misclassification is possible. To minimize this error, we selected the subjects with either RA or depression only after they were recorded as having either at least three outpatient visits reporting consistent diagnoses or one inpatient admission. It should also be noted that the NHI of Taiwan randomly samples claims from hospitals, interviews patients, and reviews medical charts to verify the accuracy of medical records^29^. Third, as data regarding RA severity were unavailable in this database, failure to adjust for the level of disability might lead to the bias. Nonetheless, the multivariate analysis applied in this study further considered the impact of several comorbidities with physical impairments, including hypertension, stroke, diabetes mellitus, heart disease, chronic kidney disease, tobacco use, alcohol dependence syndrome, and cancer. Furthermore, we performed a sensitivity analysis that was limited to only those RA subjects without comorbidities to test the robustness of our findings. The findings of this analysis revealed that RA subjects with no known comorbidities still had a higher risk of depression when compared to the general population, with adjusted HRs of 1.73 (95% CI, 1.55–1.93). Fourth, a surveillance bias may also have occurred, as those patients with either RA or depression may have been overrepresented in health-care encounters, as compared to those in the control group. To address that issue, we calculated the frequency of ambulatory care visits within the period from index date to the end of the follow-up period for each subject and further adjusted it in the multivariate regression model. The results of the reanalysis revealed that the overall adjusted HRs of depression and RA were slightly lower, but still statistically significant, with 1.63 (95% CI, 1.46–1.90) and 1.59 (95% CI, 1.33–1.72), respectively, suggesting that the number of ambulatory care visits did not appreciably impact the relationships reported earlier. Fifth, evidence derived from any observational cohort study is generally less robust than that obtained from randomized trials since cohort study designs are subject to various biases related to confounding effects. Despite our careful efforts to maintain adequate control of confounding factors, unpredictable biases could still remain if they stem from unmeasured or unknown confounders. Notwithstanding these limitations, the strengths of this study must also be acknowledged and included the immediate availability of data, the comprehensiveness of the database, and the statistical power derived from the samples’ large sizes. In addition, this retrospective 14-year cohort study allowed us to examine in detail the bidirectional relation between RA and depression that may have long latency period, and the corresponding findings could serve as a reference for future basic studies.

In conclusion, this study examined the bidirectional nature of the associations between RA and depression. Our findings suggest that RA increases the risk of subsequent depression, and the onset of depression also increases the risk of developing RA. Given the serious burdens of these two diseases on the healthcare system, recognition of the bidirectional nature of the relationships between the two diseases, along with the detection of high-risk individuals could be beneficial for healthcare providers in guiding more effective treatment strategies to improve patient care and quality of life.

## Methods

### Data Source

In order to remove financial barriers to medical care for all residents, the Taiwan Ministry of Health and Welfare launched a single-payer NHI program in 1995. At the end of 2011, >99% of Taiwan’s population was enrolled in this program^18^. Data used in this study were derived from the Longitudinal Health Insurance Database (LHID), which is a sub-data set of the National Health Insurance Research Database (NHIRD) and contains longitudinal claims data from a randomly selected samples of one million NHI beneficiaries, representing 5% of all enrollees in Taiwan. Because a multistage stratified systematic sampling method was applied, there were no statistically significant differences in sex or age between the sample group and all enrollees^29^. All data files were linked using scrambled identifications. This study was approved by the local institutional review board and ethics committee of Buddhist Dalin Tzu Chi Hospital, Taiwan (No. B10004021-1).

### Study Population

Diagnoses in the insurance claims data were coded with International Classification of Disease, 9th Revision, Clinical Modification (ICD-9-CM). In this study, two cohort analyses were conducted to investigate the bidirectional associations between RA and depression. In the first analysis, we identified adult RA patients 20–80 years of age from medical claims with a diagnosis of 714.0, and those without RA diagnosis as a control group. In the second analysis, we selected individuals 20–80 years of age with a depression diagnosis (ICD-9-CM codes of 296.2, 296.3, 300.4 or 311) and a control group without a diagnosis of depression. The subjects included in the case cohort were identified if they had at least three diagnoses in the ambulatory service or if they were admitted to the hospital with a primary diagnosis of RA (first analysis) or depression (second analysis) between 1998 and 2011. The index year was defined as the year of the first diagnosis of corresponding disease. For each case with RA (first analysis) or depression (second analysis), two control subjects were selected that were matched to the corresponding case for sex, age, and index year. In the first analysis, subjects were excluded if they had been diagnosed with depression before the first visit RA diagnosis to ascertain the temporal relationship between RA and the following onset of depression. In accordance with the same rationale, subjects with a diagnosis of RA before depression onset were excluded in the second analysis.

All subjects were then followed to the end of 2012 to obtain data on the incidence of corresponding disease within each of the two analyses. Follow-up person-years (PYs) were determined by calculating the time interval from the entry date to the earliest of one of the following: the first incidence of depression (first analysis) or RA (second analysis), the date of withdrawal from the insurance program or the date of December 31, 2012, whichever came first.

### Demographic Variables and Comorbidities

The demographic variables used in this study included age, gender, income for estimating insurance payment, and urbanization level of the subject’s residential area. Monthly incomes were divided into three levels: ≤New Taiwan Dollar (NTD) 17,880, NTD 17,881–NTD $43,900, and ≥NTD 43,901. Urbanization levels were divided into three strata: urban (levels 1–2), suburban (levels 3–4), and rural (levels 5–7) based on population density. Level 1 refers to the “most urbanized” and level 7 refers to the “least urbanized” communities^30^. Baseline comorbidities for each subjects included hypertension (ICD-9-CM 401–405), stroke (ICD-9-CM 430–438), diabetes (ICD-9-CM 250), heart disease (ICD-9-CM 410–429), chronic kidney disease (ICD-9-CM 585), tobacco use (ICD-9-CM 305.1), alcohol dependence syndrome (ICD-9-CM 303), and cancer (ICD-9-CM 140–208).

### Statistical Analysis

Intergroup difference was evaluated using the independent-samples t-test for continuous variables and the chi-square test or Fisher exact test for categorical variables. In the first analysis, we assessed the overall and sex-specific incidence of depression for RA cohorts and for non-RA cohorts. To assess the risk of developing depression associated with RA, Cox proportional hazard regression model was used to estimate the crude and adjusted hazard ratios (HRs) of developing depression among subjects with RA compared with the control group. Analytic procedure in the second analysis was identical to that applied in the first analysis. We used Cox proportional hazard regression model to assess the crude and adjusted HRs for the risk of RA in relation to depression. Furthermore, the analyses stratified by age, gender and follow-up time were conducted using Cox proportional hazards regression model to assess the bidirectional associations between these two diseases. The assumption of proportional hazards was confirmed by plotting a survival function versus a survival time graph of the log, log (survival), versus that of log survival time. All analyses were conducted using SAS version 9.3 (SAS Institute Inc., Cary, NC, USA), and at a p < 0.05 statistically significant level.

## Additional Information

**How to cite this article**: Lu, M.-C. *et al.* Bidirectional associations between rheumatoid arthritis and depression: a nationwide longitudinal study. *Sci. Rep.* **6**, 20647; doi: 10.1038/srep20647 (2016).

## Figures and Tables

**Table 1:** Demographic data and comorbidity comparison of the study subjects.

Variables	Non-RA cohort	RA cohort	*P*
	N = 15456(%)	N = 8331(%)	
Age, (years)			0.62
< = 40	2587(16.7)	1359(16.3)	
41–60	7441(48.1)	4004(48.1)	
60^+^	5428(35.1)	2968(35.6)	
Mean (Standard Deviation, SD)	54.38(14.0)	54.60(14.0)	0.24
Gender			0.12
Female	10334(66.9)	5652(67.8)	
Male	5122(33.1)	2679(32.2)	
Monthly income			0.45
Low	6673(43.2)	3592(43.1)	
Median	7942(51.4)	4253(51.1)	
High	841(5.4)	486(5.8)	
Level of urbanization			<0.001
Urban	8369(54.1)	4435(53.2)	
Suburban	2652(17.2)	1241(14.9)	
Rural	4435(28.7)	2655(31.9)	
Comorbidity			
Hypertension	3014(19.5)	2426(29.1)	<0.001
Stroke	606(3.9)	425(5.1)	<0.001
Diabetes	1412(9.1)	1162(13.9)	<0.001
Heart disease	1437(9.3)	1335(16.0)	<0.001
Chronic kidney disease	181(1.2)	160(1.9)	<0.001
Cancer	473(3.1)	269(3.2)	0.48
Alcohol dependence syndrome	7(0.0)	15(0.2)	0.001
Tobacco use	22(0.1)	14(0.2)	0.63

**Table 2:** Association of depression with RA stratified by sex, age, and follow-up years.

Variables	Non-RA cohort			RA cohort			HR (95% CI)	
	Case	PYs	Incidence	Case	PYs	Incidence	Crude	Adjusted
All	1075	120100.95	8.95	958	61075.48	15.69	1.75 (1.60–1.91)	1.69* (1.51–1.87)
Sex								
Female	834	79153.66	10.54	749	40500.41	18.49	1.76 (1.57–1.93)	1.73ξ (1.50–1.84)
<=40	123	14034.12	8.76	130	6855.65	18.96	2.16 (1.67–2.73)	2.14Υ (1.62–2.69)
41–60	427	38820.73	10.99	365	19759.52	18.47	1.68 (1.45–1.99)	1.60Υ (1.35–1.90)
>60	284	26298.81	10.79	254	13885.25	18.29	1.67 (1.42–1.92)	1.58Υ (1.37–1.82)
Male	241	40947.29	5.89	209	20575.07	10.16	1.72 (1.43–2.06)	1.65ξ (1.33–1.94)
<=40	28	7405.85	3.78	40	3548.01	11.27	2.97 (1.81–4.77)	2.76Υ (1.83–4.59)
41–60	90	17265.70	5.21	78	8682.50	8.98	1.72 (1.27–2.33)	1.59Υ (1.17–2.17)
>60	123	16275.75	7.56	91	8344.56	10.90	1.44 (1.10–1.88)	1.33Υ (1.01–1.75)
Follow–up (years)								
≦ 2	161	2481.50	64.88	195	1328.31	146.77	2.25 (1.71–2.44)	1.98* (1.43–2.15)
3–5	483	8666.64	55.73	443	5423.28	81.68	1.48 (1.34–1.73)	1.47* (1.28–1.76)
>5	431	108952.81	3.96	320	54323.89	5.89	1.50 (1.30–1.74)	1.48* (1.25–1.69)

Incidence rate is per 1,000 PYs.

^Υ^Model adjusted for urbanization level, monthly income, and comorbidity.

^ξ^Model adjusted for age, urbanization level, monthly income, and comorbidity.

^*^Model adjusted for age, gender, urbanization level, monthly income, and comorbidity.

**Table 3:** Demographic data and comorbidity comparison of the study subjects.

Variables	Non-depression cohort	Depression cohort	*P*
	N = 88,688 (%)	N = 44,727 (%)	
Age, (years)			0.60
<=40	31232(35.2)	15646(35.0)	
41–60	35230(39.7)	17774(39.7)	
60^+^	22226(25.1)	11307(25.3)	
Mean (SD)	48.16(15.39)	48.26(15.04)	0.28
Gender			0.53
Female	53810(60.7)	27218(60.9)	
Male	34878(39.3)	17509(39.1)	
Monthly income			<0.001
Low	47274(53.3)	24183(54.1)	
Median	37629(42.4)	18834(42.1)	
High	3785(4.3)	1710(3.8)	
Level of urbanization			<0.001
Urban	52169(58.8)	24135(54.0)	
Suburban	15206(17.1)	6295(14.1)	
Rural	21313(24.0)	14297(32.0)	
Comorbidity			
Hypertension	12894(17.0)	11740(26.3)	<0.001
Stroke	2432(3.2)	4435(9.9)	<0.001
Diabetes	6335(8.4)	5377(12.1)	<0.001
Heart disease	6431(8.5)	8845(19.8)	<0.001
Chronic kidney disease	672(0.9)	738(1.7)	<0.001
Cancer	1903(2.5)	2055(4.6)	<0.001
Alcohol dependence syndrome	53(0.1)	677(1.5)	<0.001
Tobacco use	173(0.2)	283(0.6)	<0.001

**Table 4:** Association of RA with depression stratified by sex, age, and follow-up years.

Variables	Non-depression cohort			Depression cohort			HR (95% CI)	
	Case	PYs	Incidence	Case	PYs	Incidence	Crude	Adjusted
All	837	692396.22	1.21	718	347046.11	2.07	1.71 (1.55–1.89)	1.65* (1.41–1.77)
Sex								
Female	664	416464.90	1.59	560	209084.69	2.68	1.68 (1.50–1.88)	1.55ξ (1.34–1.74)
<=40	90	144727.65	0.62	98	72211.49	1.36	2.18 (1.64–2.91)	1.95Υ (1.37–2.48)
41–60	317	169788.73	1.87	299	84961.55	3.51	1.89 (1.61–2.21)	1.62Υ (1.38–1.93)
60^+^	257	101948.51	2.52	163	51911.65	3.14	1.24 (1.02–1.51)	1.19Υ (0.98–1.47)
Male	173	275931.32	0.63	158	137961.42	1.15	1.83 (1.47–2.26)	1.61ξ (1.28–2.02)
<=40	20	94640.07	0.21	25	47264.23	0.52	2.50 (1.39–4.51)	2.04Υ (1.09–3.86)
41–60	71	101874.80	0.70	67	50937.94	1.32	1.89 (1.35–2.64)	1.61Υ (1.13–2.28)
60^+^	82	79416.45	1.03	66	39759.25	1.66	1.60 (1.16–2.21)	1.40Υ (0.99–1.96)
Follow-up (years)								
≦2	245	9526.52	25.72	204	4892.39	41.69	1.62 (1.35–1.96)	1.47* (1.14–1.76)
3–5	156	49293.82	3.16	153	25200.05	6.07	1.94 (1.54–2.40)	1.83* (1.61–2.19)
>5	436	633575.88	0.68	361	316953.66	1.14	1.66 (1.44–1.91)	1.58* (1.34–1.79)

Incidence rate is per 1,000 PYs.

^Υ^Model adjusted for urbanization level, monthly income, and comorbidity

^ξ^Model adjusted for age, urbanization level, monthly income, and comorbidity.

^*^Model adjusted for age, gender, urbanization level, monthly income, and comorbidity.
